# The Effects of Real-Time Interactive Multimedia Teleradiology System

**DOI:** 10.1155/2016/4126841

**Published:** 2016-05-17

**Authors:** Lilac Al-Safadi

**Affiliations:** Department of Information Technology, College of Computer and Information Sciences, King Saud University, Saudi Arabia

## Abstract

This study describes the design of a real-time interactive multimedia teleradiology system and assesses how the system is used by referring physicians in point-of-care situations and supports or hinders aspects of physician-radiologist interaction. We developed a real-time multimedia teleradiology management system that automates the transfer of images and radiologists' reports and surveyed physicians to triangulate the findings and to verify the realism and results of the experiment. The web-based survey was delivered to 150 physicians from a range of specialties. The survey was completed by 72% of physicians. Data showed a correlation between rich interactivity, satisfaction, and effectiveness. The results of our experiments suggest that real-time multimedia teleradiology systems are valued by referring physicians and may have the potential for enhancing their practice and improving patient care and highlight the critical role of multimedia technologies to provide real-time multimode interactivity in current medical care.

## 1. Introduction

Communication is an important component in healthcare. Parker and Coiera [1], Coiera [2], and Sutcliffe et al. [3] indicated that poor communication in healthcare results in high costs and undesirable clinical outcomes. Coiera [2], Coiera and Tombs [4], and Fitzpatrick et al. [5] indicated that communication between healthcare professionals is characterised as interactive, instant, and synchronous; and Varpio et al. [6] and Bezemer et al. [7] indicated that the communication between healthcare professionals is characterised as interdisciplinary and interprofessional.

*Telemedicine* is the use of electronic communication and information technologies to provide or support clinical care at a distance [8]. There are various medical specialties in which telemedicine can be used, and teleradiology is one of the most important.* Teleradiology* is the ability to transmit radiographic images, such as X-rays, computed tomographic images (CT) and magnetic resonance imaging (MRI) from a remote location to a radiologist's location to facilitate accurate diagnosis. The importance of teleradiology lies in the improvement of medical services and in the availability of quality medicine, mostly in distant areas [9]. In comparison with medical online communities, teleradiology currently has a slow image transfer, lacks a feedback mechanism, and is limited to a certain number of participants [10–14]. In addition, clinicians find that teleradiology demonstrates a fragmented and interrupted experience because text, images, and speech are delivered on different channels and modes.

Research on multimedia shows that humans use both text and images to construct coherent mental knowledge representations [15–19]. This can be achieved by presenting words and images together and in a well-designed and integrated manner. The construction of coherent mental representations supports learning and decision-making [18, 19]. Hence, although medical images play an important role in making good decisions on the diagnosis of particular case, medical consultation between physicians and radiologists would become more meaningful if tools were available that not only displayed medical images on their consoles but also supported the integration of written words, spoken words, and images.

Advances in multimedia technologies and applications and the availability of high-speed bandwidth internet have increased the utilisation of multimedia in healthcare such as supplementary materials to demonstrate the ideal practice of procedures [20, 21] and resources to help students prepare for the clinical examinations [22, 23] and to orient residents and students to the operations of hospital departments, and it has also been integrated into electronic medical records [24]. A number of works extended existing teleradiology systems with multimedia components to support interaction [25, 26], collaboration [27], or advanced visualisation [28]. However, the multimedia components have not been efficiently integrated into a single healthcare system [29], and the value of integration for effective interaction and knowledge exchange between referring physicians and radiologists has not been tested.

In this paper we present a real-time multimedia interactive teleradiology management system (MTMS) to support physicians' diagnoses. MTMS provides real-time interaction using several modes (video, audio, and text), and offers a display of radiographic images, image annotation tools, an archiving and retrieving system, real-time interaction, and an audio reporting feature. This is achieved through full integration and controllability of advanced technologies in multimedia—video and audio transmission, image compression and processing, and voice recognition, all in a single application and in real time. The developed system is particularly useful in providing immediate diagnosis for a remote medical care center. The system provides remote consultation in such a way that both the referring physician and radiologist can review the same image and discuss it with each other by the dialog window using various modes.

### 1.1. Problem Definition and Goals

Local healthcare institutions, in particular, primary and secondary care centers, provide lower quality healthcare services because of the lack of experienced staff and adequate technical resources [30]. This means patients have to be transferred to hospitals with appropriate facilities for diagnosis and treatment. These transfers are costly and time-consuming [31].

Traditional, text-only radiology reports are the major, and often only, means of communication between radiologists and their referring clinicians [32]. Although advances in image display and distribution have made some radiology images more readily available to referring physicians, the increase in imaging data generated increases the need for direct involvement of experts in real time [33].

A number of solutions have been provided to connect referring physicians in remote areas with radiologists, as will be described in Section 1.2. However, many physicians are skeptical about the value of remote consultation and remain in favour of face-to-face consultation.

In order to respond to the physicians' concerns, we developed and tested a real-time interactive multimedia teleradiology system. The main goal of MTMS is to serve less well-equipped medical institutions by supporting teleradiology with a number of integrated multimedia tools, in order to improve the quality and efficiency of diagnosis in regular and emergency cases. Our main contribution in this paper is to (1) leverage multimedia technological advances to improve the interaction and achievement of referring physicians, (2) evaluate the performance of a multilayered integration-based architectural design, and (3) quantify referring physicians' perceived value of real-time multimode interaction with radiologists in improving radiologists' satisfaction and in improving their effectiveness.

### 1.2. Background

A number of teleradiology management systems exist for commercial use. XRAYLINE (http://www.xrayline.com/) is an online portal that allows physicians and radiology staff to share medical images and preview online radiology studies. The following services are provided by XRAYLINE: the ability to view images and report online, preview exam results for referred patients, access images at any time from personal computers with a browser and internet connection, download exams, and access local personal archive servers, which can store, forward, query, and retrieve exams from different modalities or other servers.

Miner Miracles Ltd. (http://www.medicexchange.com/) is a company that provides system management, integration, and development for general business and healthcare industries. It provides a management system in digital radiology and teleradiology, security network and maintenance, consultancy, and help desk assistance tracking. Miner Miracles Ltd. provides the following: image viewer software for displaying and manipulating images, a reporting cycle with dictation, and transmission of images.

MxiPACS [34] provides teleradiology management system services. It provides several services such as image viewing and sending of images. The features also include manipulation tools and the facility to download image exams and reports.

These commercial systems are based on the store and forward (S&F) method, which involves sending data (including medical images, lab work, etc.) to the physician, who can assess it at a convenient time. This method does not require both parties to be present at the same time. It is a consultation in an asynchronous mode rather than real-time mode [8].

Various researchers have focused on interactive televideo (ITV) methodology in implementing teleradiology [25–29, 35]. ITV involves video conferencing and more advanced technology. Physicians can be present in primary care on one side and radiologists must be present at the same time on the other side of the encounter. It is also called the real-time method and simulates real-world interaction with patients [8]. However, to our knowledge, no research has evaluated the value of ITV teleradiology systems as perceived by referring physicians. This experimental study is the first to assess the effects of real-time multimode interactive teleradiology to physicians' satisfaction and patient care in Saudi Arabia.

## 2. System Description

MTMS is designed to test a number of assumptions relevant to physician-radiologist interaction extracted from various research papers.

1. Physicians desire accurate and timely reports over those that are well-organised [36, 37].
2. Real-time consultation between physician and radiologist is essential [38].
3. Visual, audio, and textual information needs to be accessed synchronously to improve patient care [39].
4. Enhanced personal interaction increases physician satisfaction [38, 40]. This can be achieved through rich multimodal interaction.
5. Multimode interaction increases physician effectiveness in clinical performance [39, 40].
6. Access to previous examinations is needed [38].

**Figure 1:**
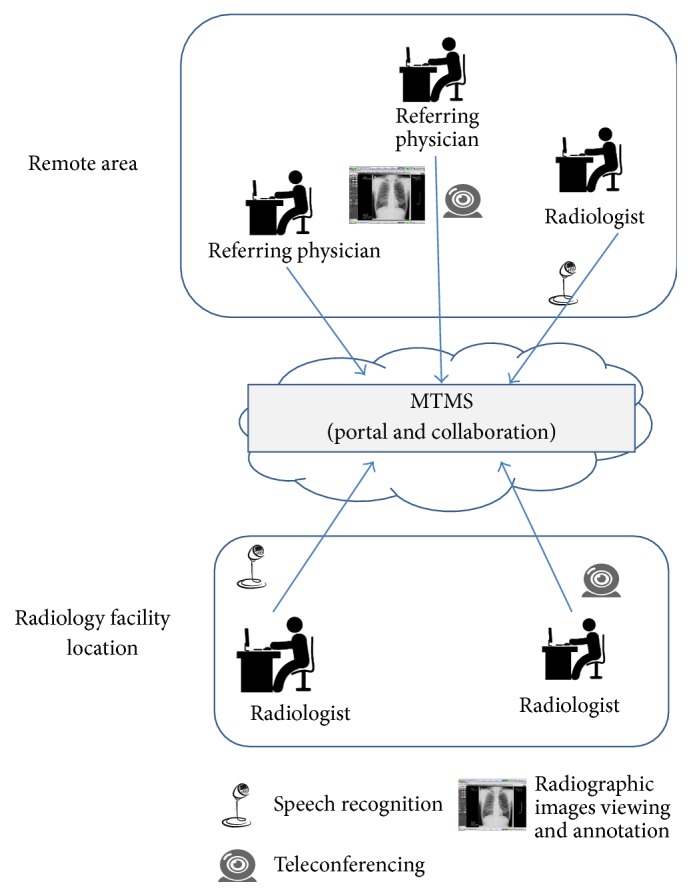
MTMS system description.

The online MTMS provides an environment and platform to support referring physicians and clinicians in remote areas or less well-equipped medical care centers (Figure 1). MTMS provides two categories of features: medical case management and user management. The medical case management system provides the following features: establish a medical case, add radiographic images and reports to the medical case, annotate images using text and shapes to highlight interesting areas in real time, provide real-time interaction using various media modes, compress and archive medical cases, view cases with different levels of granularity, search and browse medical cases using advanced search criteria, develop radiology reports and document conversation using voice recognition, assign and unassign medical case to a radiologist in a radiology facility location, and view X-ray images in a special Digital Imaging and Communications in Medicine (DICOM) viewer [41].

### 2.1. Architectural Design

**Figure 2:**
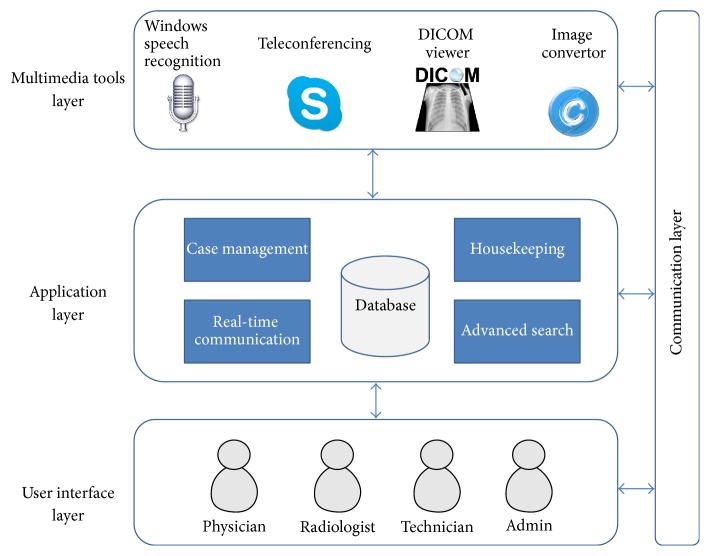
MTMS multilayer integration-based architectural design.

The architectural design satisfies the functional and nonfunctional system requirements. It establishes the basic structural framework that identifies the major components of a system and communication between these components. This project was developed using a multilayer integration-based architecture (Figure 2). The multilayers are the communication layer, application layer, multimedia tool layers, and user interface layer.

The tool integration architecture helps to optimise the overall functionality of the system by using prebuilt applications or tools across a network. In addition, it allows quick development of the system and delivers a more efficient and accessible application. MTMS integrates four multimedia supporting tools to achieve the goals and objectives of this system.

1. Image converter: software that converts the radiographic images from DICOM format (.dcm) to bitmap format (.bmp) and vice versa. This enables the radiographic images to be transferred, received, and stored in a patient database or other archives.
2. A DICOM viewer: an application to process the radiographic images in DICOM format with high resolution. It enables the radiologist to view the case images in an external DICOM viewer with high resolution imagery and extra features like zoom, print, brightness/contrast control, and so forth.
3. Speech recognition: a software that recognises the voice and converts it to text. It gives the radiologist the opportunity to develop a report automatically and attach it to a medical case using voice and hence reduces the user's keystrokes and the time and effort involved in typing.
4. Teleconferencing: an application with many features like video call, audio call, and instant messaging. It provides the referring physician and the radiologist with a tool for real-time communication so they can discuss medical cases.

### 2.2. Implementation

To experiment and test the introduced concept, a prototype was developed using C# programming language and Microsoft  .Net framework 3.5 as part of a graduation project.  .NET framework was chosen for its large library and its support for several programming languages, which allows language interoperability. In addition to this, the  .NET Framework's Base Class Library provides integration with other outsourced libraries.  .NET framework is fully compatible and runs successfully on machines running Microsoft Windows operating systems (Windows XP, Vista, and 7). A Microsoft SQL Server 2008 was used to run the database and store the medical cases.

System integration is about successfully putting together parts of systems or subsystems to work as one unit to perform what the system was intended to do. MTMS joins the functions of a set of subsystems or software through integrating a number of tools to produce a unified system that supports the requirements of the system. MTMS takes the advantages and power of existing tools, which saves time and effort in the implementation phase. MTMS integrates Skype v5.5 (http://www.skype.com/) to gain the advantage of features that Skype provides, such as group teleconferencing and real-time teleconferencing. In addition, it uses MICRODICOM (http://www.microdicom.com/), an image viewer that allows users to manipulate and view radiographic images. The Dicom2 Converter (http://www.barre.nom.fr/medical/dicom2/) converts DICOM images to portable compressed format and vice versa. This is required to integrate the radiographic images in the displayed form and to enable their storage in the database. Microsoft Speech Recognition allows radiologists to record medical reports easily and faster.

**Figure 3:**
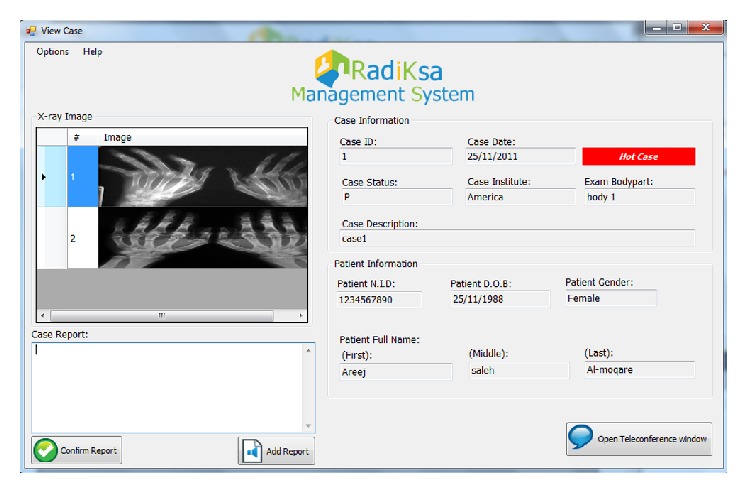
View case screen for physicians—prototype.

Figure 3 shows one of the physician's screens. The screen is composed of four main sections: the radiographic images display area, the patient's information, the case report area, and the case details.

## 3. Testing and Results

A variety of experiments and tests were carried out in Saudi Arabia from June 2014 to January 2015 on the system to explore its functionality and to identify any problems. The experiments were performed to show (1) how the system performs technically and (2) how the end-users perceive the value of the system, in particular, to identify referring physicians' preferences about integrating multimedia tools with teleradiology systems to facilitate real-time and enhanced interaction, and to quantify their perceived value of this system compared with text-based communication alone.

### 3.1. User Interface Testing

A user observation technique was used for user interface testing. This involved watching and listening carefully to users as they worked with the product. Two categories of users participated in the user interface test: eight physicians and three radiologists. One hundred per cent were female with the majority having a range of 3–5 years of experience (73%). Six user observation sessions took place. Users were asked to perform a total of 20 tasks selected to test the functional and nonfunctional system requirements of the system. The following tasks were performed: conduct simple and advanced search for a medical case, retrieve archived medical case, view the medical case at different granularities, add a medical case, upload a number of radiographic images, enter a radiology report, establish real-time interaction using voice, video, and text, and support interaction with annotation tools.

Overall results were acceptable as 90% of the tasks were accomplished within the expected time, with an 18% error rate. Corrective actions were taken to reduce the errors made. The majority of participants viewed each case between one to three times and spent sufficient time on the content. Seventy-eight per cent agreed that the quality of images was satisfactory, 60% agreed that the voice was clear, 65% agreed that the design of the system was consistent and allowed easy navigation, and 63% of the participants thought they could understand the procedure by themselves without any further clarification. The overall familiarity with the system helped in decreasing the time required and errors made in performing tasks.

### 3.2. Performance Testing of Speech Recognition

As regards testing the performance of the speech recognition system, ten radiology reports were tested. Language and size of the report were the only criteria for report selection. On average a report of 350 words was developed in 2.7 minutes and with an average of eight errors, giving 97.8% accuracy. This was quite acceptable compared with the time and effort saved. Software training increases the accuracy of speech recognition results.

### 3.3. User Acceptance Testing

To quantify the referring physicians' perceived value of multimedia technologies and to test the assumptions that referring physicians would value real-time rich interactivity with radiologists, a survey was designed. Physicians were provided with a full description of the system and a link to try the system. They briefly analysed a patient case to initiate a consultation with a radiologist. The patient cases were delivered in two different modes: text-only reports and full integrated mode (text, verbal, image, and real-time interaction). A web-based survey was developed and administered to a test sample of three physicians and then revised. Experts evaluated the survey for clarity and comprehensiveness. The survey included 25 questions and open-ended comments on advantages and disadvantages of the system and difficulties faced while using it. The different sections in the survey covered (1) participants' demographics; (2) physician satisfaction with the multimode real-time interaction; and (3) contribution of multimode real-time interaction to effectiveness questionnaires.

Physicians were asked to rate their agreement concerning their satisfaction and effectiveness by the use of a Likert scale. Assuming 30 participants from five different hospitals in Saudi Arabia, the revised survey was delivered to 150 physicians from a range of specialties via email, with a 20% drop-out calculated rate. The survey was available for 60 days. Missing responses were excluded from analyses, and *n* was calculated based on the actual number of respondents in the sample. Participants' email addresses were pulled from the hospitals' directories. A reminder message was sent by email every week or two to all participants and personal visits were conducted to encourage participation. Seventy-two per cent of physicians completed the survey. The mean age of respondent was 25 (0.5) years, with the majority of them being female (69%). The majority of physicians accessed the system from home (60%).

### 3.4. Ethical Issues

The study request was reviewed by the hospital management who concluded that they did not need special approval as this was nonclinical trial that did not investigate health outcomes. Implied consent was obtained from test participants who completed and returned questionnaires.

### 3.5. Results

Data analyses were performed using Excel 2010 programme. A *p* value of less than 0.05 was considered significant. Overall, physicians were satisfied with a system that provided real-time multimodal communication with radiologists (3.9 + 0.56). The item that received the highest rank in the satisfaction questionnaire was “multimedia components enhance understanding of a text-based report” (4.23 + 0.9). Physicians also reported high effectiveness for improving patient referral and healthcare delivery. The overall mean effectiveness out of 5-point Likert-type scale was 4.38 (0.42). Additionally, the integrated model of interaction achieved a significantly higher score to the grades of the text-only reports (*p* < 0.05).

#### 3.5.1. The Effect on Effectiveness

Almost 70% of physicians provided positive comments to the qualitative questions related to the effect of real-time multimode interaction on the physicians' effectiveness. According to physicians, the real-time multimedia consultation would significantly improve patient safety and care, decrease their stress in making decisions from a distance, enhance understanding of medical images, improve the quality of interactions with radiologists, and improve patient care as a whole (70%, *n* = 72).

**Figure 4:**
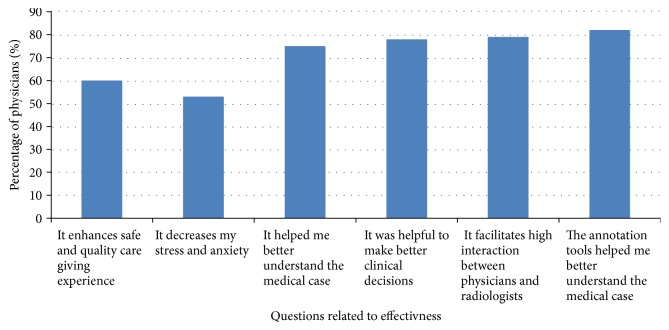
The effect of real-time multimode interaction on effectiveness.

The online annotation tools were also reported as a very valuable interaction resource by 82% of physicians (*n* = 83). The integration of annotation tools with real-time video conferencing was very useful in decreasing physicians' anxiety and improving their overall clinical performance. Physician comments included the following: “In the clinic, I found it easier to recall and relate to the annotated image instead of the description in the text-only report” and “MTMS helped me know exactly the medical case I have in hand.” In addition, 74% agreed that recording communication for later access was one of the required functions.  Figure 4 shows the percentage of physicians who agreed and strongly agreed with each separate item on the positive effect of real-time multimode interaction on effectiveness.

#### 3.5.2. The Effect on Satisfaction

**Figure 5:**
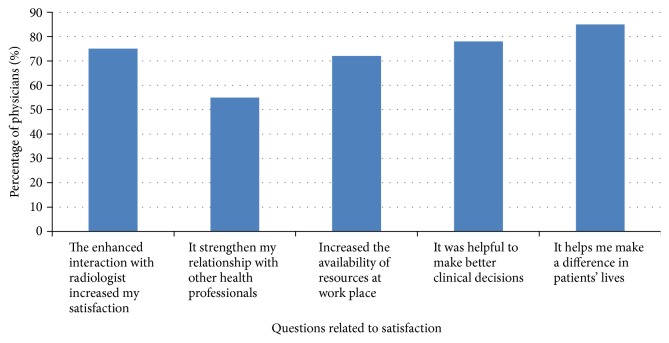
The effect of real-time multimode interaction on satisfaction.

Overall, 75% of physicians provided positive comments to the satisfaction questions. Physicians' satisfaction measurements were based on Konrad et al.'s physicians' satisfaction criteria [42] and included relationships with other health professionals, the availability of resources at work place, satisfaction that comes from doing the job, and satisfaction with being able to make a difference in patients' lives. Physician comments included the following: “MTMS will help in making a difference for patients who live in less developed areas.” Figure 5 shows the percentages of physicians who agreed and strongly agreed with each separate item on the positive effect of real-time multimode interaction on satisfaction.

Although many physicians were enthusiastic about the idea of real-time multimedia interaction, several also expressed concerns about the time required to download images, the precision of the speech recognition, and the clarity of real-time interactive communication across data networks.

Overall, however, the responses support our design assumptions and the idea that there is value in providing integrated multimedia tools as part of a teleradiology management system that supports real-time interaction with enriched documentation features.

The results of our study suggest that real-time multimedia teleradiology systems have a high perceived value amongst referring physicians and may have the potential for enhancing the practice of referring physicians and improving patient care.

#### 3.5.3. Radiologists' Perspective

Radiologists also reported their experience using a questionnaire. From the radiologist's perspective, the use of real-time multimodal interaction helped provide rich interaction experience with referring physicians. In addition, radiologists have captured 24% potential inappropriate performance by physicians due to misinterpretation of the text-only radiology reports. These were mainly due to insufficient description of the radiographic images in the text reports and overall clarity of received reports. The radiologists, as well, valued the recorded communication for later access and mentioned that this function can be used as a source for learning and research (72%): “Recorded interaction will reinforce the learning process and facilitate transition to practice” and “the best evidence-based reference of consultation received.”

## 4. Discussion

Because of the limited resources and technologies in medical care units in rural areas, creating real-time access to radiology facility locations using multimedia technologies should be a high priority in terms of enhancing the practice and satisfaction of referring physicians, improving patient care, and emphasising the critical role radiology plays in medical care.

The developed prototype has been designed and implemented for patient treatment to test assumptions relevant to teleradiology. These assumptions were related to the need for (1) real-time consultation between physician and radiologist, (2) access to visual, audio, and textual information synchronously, (3) rich multimodal interaction, and (4) access to previous examinations.

The unified integration of multimedia components in a single computer provides an efficient means to investigate the above assumptions. The software implementation using an integration model of existing off-the-shelf software offers a compact design at a low cost and with ease of use.

Although this project was led by specialists in information technology, the interdisciplinary collaboration between healthcare providers, faculty members, multimedia experts, IT experts, and administrators was a key to the project success.

Experimental tests analysed the technical aspects of the implemented systems and showed that the system could be run with acceptable error. Saving caregivers communication for later access was one of the required functions. Since Skype video calling does not support storage of recorded conversation, other teleconferencing systems will be considered. In addition, in the future, the system will provide access to patients as users to enable them to track their own cases in the system and communicate with their physician at a distance.

The study described physicians' responses to an interactive consultation environment based on the integration of a number of multimedia applications and technologies. The results showed a high level of satisfaction, indicating the effectiveness of the new consultation strategies. The use of real-time interaction decreased physician anxiety, increased physician satisfaction, and improved their ability to succeed in their work. Physicians valued the high interaction and indicated that the interaction and multimedia integration of information can promote safe and high quality care giving experience.

**Figure 6:**
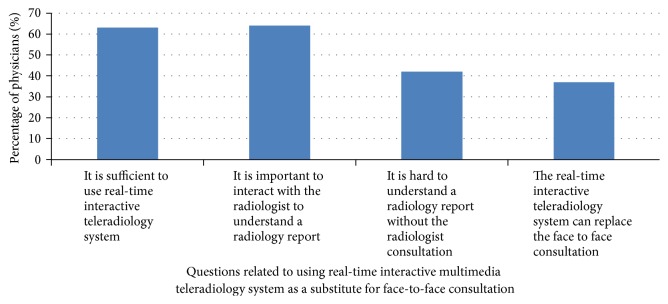
Real-time multimode interactive teleradiology system as a substitute for radiologist consultation.

Almost half of the referring physicians (*n* = 46) still think that face-to-face consultation is more effective and preferred to use MTMS as a supplement rather than a replacement for face-to-face consultation. Physicians thought that face-to-face interaction built more trust, clearly shared understanding, and fostered a greater engagement from radiologists. Physician comments included “patient diagnosis is a sensitive task where it cannot tolerate any misinterpretation.” Figure 6 shows the percentage of physicians who agreed and strongly agreed on using a real-time interactive multimedia teleradiology system as a substitute for face-to-face consultation. However, with the new technologically savvy generation, the pressure exerted by advances in technology, and radiologist and physician shortages along with patient demand for flexible patient care, the momentum of multimedia technology growth is likely to continue to increase in the future.

### 4.1. Limitations

The findings still need to be interpreted and generalised with respect to several limitations. First, more testing should take place on areas such as the overall performance of the system and the quality of images. Second, the participants were physicians in hospitals in the big cities of Saudi Arabia. We need to test the system with participants in rural areas as they represent a group with less advanced medical and technological experience. Third, the study was conducted in an experiment setting where the environment was relatively quiet compared to the hectic, noisy, interrupted, and chaotic context of real clinical communication. In a real clinical setting, it remains unclear to what extend physicians are willing to use advanced communication tools and if the integration of multimedia technologies can contribute to cognitive overload using multiple channels simultaneously. Fourth, we have not reached an answer on how the multimedia components help physicians make fewer errors. Follow-up studies need to support the sustainability of the above preliminary results.

## 5. Conclusions

Real-time interaction is fundamental for healthcare. The use of multimedia with an interactive consultation environment is a valuable strategy that yields a better diagnosis and clinician satisfaction. The creation and delivery of a real-time interactive consultation environment are a complex process that should be carefully designed. An integration-based model allows quick development of the system and delivers a more efficient and accessible application. The design of the system was based on a number of assumptions relevant to physician-radiologist interaction. The results of the experiments support the design assumptions and show that there is value in providing integrated multimedia tools as part of a teleradiology management system to support real-time communication with enriched documentation and interaction features.
